# “Our children are the forgotten children”: lived experiences of COVID-19 asymptomatic testing in young people with special educational needs and disabilities and those who care for them

**DOI:** 10.1186/s12889-026-28743-w

**Published:** 2026-07-28

**Authors:** Aimee Harragan, Lisa Thorpe, Holly Carter, Richard Amlôt, Charlotte Robin

**Affiliations:** 1 Behavioural Science and Insights Unit, UK Health Security Agency, Suite 3B, 3rd Floor, Cunard Building, Water Street, Liverpool, L3 1DS UK; 2 Behavioural Science and Insights Unit, UK Health Security Agency, Leeds, UK; 3 Behavioural Science and Insights Unit, UK Health Security Agency, Salisbury, UK

**Keywords:** Special Educational Needs and Disabilities (SEND), COVID-19 testing, Equitable access, Lived experience, Public health, Health protection.

## Abstract

**Background:**

The impact of the COVID-19 pandemic was felt disproportionately by people with Special Educational Needs and Disabilities (SEND), with a substantially higher mortality rate compared with the general population. Regular testing was an important part of the public health response to reduce infections, however many people with SEND were unable to access COVID-19 testing. Our aim was to understand SEND experiences of and barriers to COVID-19 testing.

**Method:**

We conducted focus groups with young people with SEND and those who care for them to explore their experiences of COVID-19 testing. Thematic analysis was used to identify challenges encountered and how they were navigated.

**Results:**

Participants reported a systemic misunderstanding of SEND needs underpinned experiences of disempowerment, being overlooked, and feeling that needs had been unmet during the pandemic. This sits in contrast with the considerable expertise that parents and families exhibit in understanding the needs of their children with SEND. Participants expressed feeling their opportunity to engage in testing was restricted through a misunderstanding of how their physical and communication needs may differ from the wider population.

**Conclusions:**

Our findings highlight how a misunderstanding of SEND needs and negative impacts of attempts to engage in testing despite considerable barriers, left SEND communities feeling forgotten during the pandemic. We propose the following recommendations: harnessing these insights in future public health response measures, using robust behavioural and social science approaches; acknowledgment of participant expertise of experience through co-produced public health guidance, crucially reflecting the diversity of SEND; development of more inclusive guidance to testing practice which supports equitable access for those with SEND and guides face-to-face practitioners in providing this support.

**Supplementary Information:**

The online version contains supplementary material available at https://doi.org/10.1186/s12889-026-28743-w.

## Background

The impact of the COVID-19 pandemic changed everyday life for most communities around the globe. Public health measures introduced by governments resulted in pervasive societal changes, including closure of schools, workplaces, and other institutions. Individuals were required to adjust to new routines of social distancing, mask wearing, isolation and regular testing.

For people with Special Educational Needs and Disabilities (SEND), systemic barriers to healthcare have resulted in lower engagement with preventive healthcare [1] and several barriers to engaging with healthcare services in other contexts have previously been identified. These include physical barriers, such as a lack of appropriate facilities or environments [2], specifically sensory-stimulating waiting rooms [3, 4] and lack of time for appointments [2]. Inadequate SEND-specific knowledge and low confidence in healthcare providers have also been identified as barriers to accessing healthcare [2, 4]. This has been shown to result in feelings of needs being misunderstood and marginalisation, particularly from mothers of young children [4, 5]. The systemic barriers are a result of the stigma, discrimination and marginalisation people with SEND often experience [5–7].

The collateral impacts of public health measures were felt particularly profoundly for people with SEND, where these systemic barriers to engaging with healthcare are already embedded. In addition, the abrupt disruption to daily lives was felt more keenly for those with SEND, as establishing new routines can be challenging; time and space is needed to enable them to feel safe [8–11]. These impacts sat against the troubling landscape of the disproportionate health impact of the pandemic itself on those with SEND [8, 12, 13], which in the United Kingdom (UK), resulted in a mortality rate 6.3 times higher than the general population [14].

To date, research on COVID-19 and the SEND community focuses on the wider impact of the pandemic; there is limited research on SEND engagement with COVID-19 interventions, specifically testing. However, where research has been conducted on other interventions, it demonstrates how the pandemic amplified existing barriers for access to healthcare. For example, sensory sensitivity has been identified as a contributor to vaccine hesitancy for people who are neurodivergent; the loud, fast-paced environments in which COVID-19 vaccinations were often offered as part of mass vaccination drives resulted in low vaccine confidence for those in the Neuro-Diverse (ND) community [15].

In England, regular testing was crucial to limiting the spread of the virus and was used to ensure that the transmission of virus was kept to a minimum [16]. From April 2021, regular COVID-19 testing was available for everyone in England. The main types of tests used were Lateral Flow Device (LFD) and Polymerase Chain Reaction (PCR) tests. However, many parents of children with SEND had reported that due their child’s complex needs, they were not able to engage in the use of these testing methods and used local media to voice their need for a testing alternative to PCR and LFDs which would be suitable for their child [17]. Barriers associated with LFD and PCR testing options included the invasiveness of the nasal swab and the need to restrain their child [17, 18]. The aim of our study was to understand the SEND community’s experiences of COVID-19 testing, particularly any challenges they encountered. Specifically important to this research was capturing this experience and its impact in the context of the lived experience of those with SEND and their families.

## Methods

### Study design

The approach of this project adopted an appreciation of the importance of consulting children and young people as actors with agency in their own lives and decisions that are relevant to them [13, 19–21]. This has been achieved by including young people with SEND as participants guided by VOICE principles [21, 22], as well as including those who care for them. The design of this project also appreciates the social model of disability and how this interacts with SEND categorisations and experiences [22–24]. It also takes heed of the WHO’s International Classification of Functioning, Disability and Health which adopts a biopsychosocial approach to health, appreciating environmental and contextual elements of health and disability and how they are experienced [25]. Whilst the Departments of Education and of Health set out a definition of special educational needs as: “a child or young person has SEN if they have a learning difficulty or disability which calls for special educational provision to be made for him or her” [26], definitions of SEND have been challenged. This is on account of the broad and complex spectrum of needs and people encompassed within the term, which can lead to a compounding of oversight of needs, particularly when a diversity of people with individual needs are categorised under such an umbrella [8, 27, 28]. Whilst recognising these challenges, the term SEND has been used in this paper to align with its current use in policy, education and everyday conversations, to ensure the recommendations of this paper resonate to widest possible audience using terms currently well understood.

To gather data to explore lived experiences of COVID-19 testing amongst young people with SEND, focus groups were selected as a method that could facilitate a rich discussion where participants could share in both individual and group experience [22, 29–31]. Coates and Vickerman offer several recommendations to follow when engaging young people with special educational needs in consultations. These principles are summarised as “VOICE (versatile, opportunity, inclusive, creative, empower): researchers and practitioners need to be versatile in their approach; children of all abilities should also be given the opportunity to participate in research; research must be inclusive; researchers should therefore be creative; researchers should seek to empower children they engage with” [21, 22]. While Coates and Vickerman focus particularly on physical education experience, the principles they outline regarding engagement of young people with special educational needs can be applied to other topic areas. This is on account of the fact that embracing the ideals of ‘VOICE’ allows for children and young people to be empowered with the opportunity to be consulted on matters which concern and impact them, irrespective of context. Our study followed these principles by developing Easy Read [32] information sheets to enable participants with SEND who needed extra support to engage in this information directly or to support parents in having conversations with their children about agreement to participate in the project, depending on their needs (Supplementary File 2) [21, 30]., Our research team also created other visual engagement materials such as storyboard and mood cards and included trusted adults in focus groups. Our researchers remained nimble in their approach and factored in approach protocol in case of distress in participants. Further details in the below data collection section. Our study aimed to capture a deeper understanding of the SEND community’s experience of COVID-19 testing, particularly exploring any challenges encountered when engaging with testing guidance and practice.

### Data collection

Ten semi-structured focus groups were completed: two focus groups with young people (aged 11–19 years) with SEND (*n* = 9); eight focus groups with parents or guardians of children with SEND (*n* = 20). The focus groups with young people with SEND took place in person at pre-arranged local venues. The focus groups with parents or carers were conducted online via Microsoft Teams between, with all sessions taking place between April and May 2022. Building on the literature behind empowering all children and young people to contribute their views [22, 30, 33], whilst recognising that focus groups can have limitations when conducting research with young people with SEND [28], particular attention was paid to the ways in which qualitative methods could be made as accessible as possible for our participants. To make the focus groups inclusive to all, flash cards (see Supplementary File 1) were available to allow individuals with SEND to express themselves non-verbally, if needed, using a storyboard and mood cards. Instead of questions, a story using fictional characters, based on COVID-19 testing was presented visually and participants were invited to fill in sections of the story using the mood cards available. These ‘visualisations’ of the research were intended to support all participants with SEND to engage according to their needs or preferences [31, 34]. For the focus groups with individuals with SEND, a trusted and suitably qualified adult was present for the discussion to follow safeguarding guidelines and to ensure that the participants did not become distressed at any time throughout the discussion.

### Participants

Participants with SEND (*n* = 9) were aged between 11 and 19 years. Parent participants (*n* = 20) had children between the ages of 5–17 years. All participants with SEND self-reported a primary diagnosis of Autism Spectrum Disorder (ASD), with 2 reporting an additional learning disability (LD) and 1 reporting Attention Deficit Hyperactivity Disorder (ADHD). The parent participants reported a range of diagnoses for their children, including ASD, ADHD, LD, social, emotional and communication difficulties, sensory processing disorder and physical disabilities include deafness and blindness.

### Recruitment

Participants were recruited through local authority contacts, charitable organisations and online forums based in the North of England. Participating gatekeeper organisations supported young people (aged 11–21 years) across the spectrum of SEND, including ASD. Other organisations included dedicated support groups for the parents and carers of children and young people with SEND. Gatekeeper organisations were provided with study recruitment information, which they shared within their networks. As this was responsive work conducted during the pandemic, we did not aim to achieve data saturation. Instead, we recruited as many participants as possible through existing networks, and our analysis focused on ensuring that their experiences were meaningfully and comprehensively represented.

### Analysis

The focus groups were audio-recorded, either using a Dictaphone or by recording the session on Microsoft Teams. These recordings were transcribed verbatim by a third party and NVivo was used to store and analyse the transcripts. Data from the focus groups with individuals with SEND and the focus groups with parents were analysed alongside one another. Inductive thematic analysis was conducted to identify key themes and two iterations of a coding framework, beginning with open-coding, were developed collaboratively by the team of two behavioural scientists [31, 35, 36]. To develop the first coding framework, 30% of transcripts were coded independently by the research team, who then met to discuss codes and reach a consensus on the first iteration of the framework. This framework was then applied to the remaining data and to ensure a thorough understanding and appreciation of the data, two members of the team collaborated during analysis, developing the second coding framework iteratively, based on evaluation of the findings. Due to staff changes, the data were collected and analysed by different members of the research team, the team member who collected the data left the team, meaning two other team members analysed the data collected.

### Ethics

Ethical approval was granted by UK Health Security Agency’s (UKHSA) Research Ethics and Governance Group (REGG) based on submission of a study protocol (including information sheets and consent forms), focus group topic guides and supporting methodological materials including the mood cards and storyboards [36]. Participant information sheets were provided to all participants. This included Easy Read [37] versions to enable participants with SEND who needed extra support to engage in this information directly (Supplementary File 2) [parsons21, nind30]. Consent was sought from all participants including, where relevant, from parents and guardians for young people aged under 16 years, and for those with SEND who needed them, accessible study materials were provided to facilitate their engagement with the consent process. In the case of the focus groups with young people with SEND a trusted and suitably qualified adult was present for the discussions to follow safeguarding guidelines of the gatekeeper organisations and to ensure no participants became distressed at any time throughout the discussion.

### Reflexivity

The member of the research team who conducted the data collection for this project had previous experience of working as teacher, with some experience of working with pupils with SEND. Another member of the research team involved in the design and the analysis of the research had previous qualitative research experience with adults with SEND in a local authority setting. These experiences helped to strengthen the appreciation of the need for flexibility in both design and conduct of the project, such as understanding that young people with SEND are not a homogenous group, and factoring in the need to pivot approaches to work best with the needs of the participants. The background of the researchers’ experience supported the approach to the coding of the data in order to capture the nuance of experience for this community [31].

## Results

In what follows, we explore two key themes of the results of discussions with our participants: ‘perceived negative impacts’ and ‘(mis)understanding of needs’. The theme of ‘perceived negative impacts’ relates to the adverse elements of COVID-19 testing reported by both young people with SEND and parents of children and young people with SEND. Within this key theme, we further unpack four subthemes: ‘physical challenges of administering tests’, ‘parental guilt’, ‘lasting impacts’ and ‘illusion of choice’. The key theme of ‘(mis)understanding of needs’ relates to people with SEND and those who care for them feeling that their opportunity to engage in testing had been hampered and undermined by barriers underpinned by a misunderstanding of how their needs may differ from those of the wider population. These experiences amplified how parents and carers have a deep understanding of how and why the testing offer needs to be adapted and therefore more accessible. As such, the theme of (mis)understanding of needs includes the subthemes of unmet needs in terms of physical and communication needs: ‘unmet physical needs’, ‘unmet communication needs’, but also the subthemes related to being experts in their own experience: ‘experts in their own experience’, ‘importance of routine’, ‘needing time to adjust’, ‘parents filling capability gap’ and ‘parents know best’.

### Perceived negative impacts

#### Physical challenges administering tests

Perceived negative impacts of the experiences of testing were expressed from the perspectives of both young people with SEND and their parents. Highlighted in discussions across all focus groups, the difficulties both parents and young people experienced when performing PCR or LFD tests related to the ways in which the features of the tests conflicted with the sometimes complex needs of the users, resulting in both physical and emotional negative effects. Parents reported many occasions where they have felt the need to physically restrain their children to conduct a PCR or LFD test as their child, who may have a spectrum of additional needs, tried to resist the throat or nasal swab:

“What happens is, we chase him round the house with a sensory blanket, we grab him, wrestle him to the ground, his dad wrestles him to the ground, I get the swab out, shove it up his hooter [nose] whilst pinning his head down, and that’s how we test him” (Parent Focus group 1).

The resistance was reported to be rooted in the physical discomfort or pain of the nasal or throat swabs. This was compounded by, in some cases, a lack of understanding of the need for or purpose of the test, particularly when children were young or approaching the test prompted *“meltdowns” (Parents Focus Group 7).* When asked to describe their experience of COVID-19 testing, participants with SEND said: “*painful and it still is”*,* “awful”*,* “agony”*,* “I think up the nose is more painful” (SEND Focus Group 1 and 2).*

#### Parental guilt

This use of restraint resulted in feelings of parental guilt and this guilt was expressed in conflict to the moral decision to follow government guidelines:

“We were definitely caught over a barrel. We had no choice in all this, they’re our children. And I’ve got to live with mine, it’s the guilt, and I hate that for him” (Parent Focus Group 7).

“But it’s frightening when they’re restraining him. Like I said, I had to have somebody holding his head and his body was strapped in his chair, and I’m still terrified that I’m going to take his brain out. You know what I mean? It’s horrendous, isn’t it?” (Parents Focus Group 7).

“Before COVID if someone did that to you it was classed as abuse. But now it’s fine because the government said so” (Parents Focus Group 7).

These experiences reportedly impacted the wellbeing of both parents, children and young people.

#### Lasting impacts

The negative emotional impact testing and anticipation of testing has had on young people with SEND because of the need to use restraint, was reported to have had a lasting impact on safe spaces, trust and relationships. The fraught emotional experiences, reported as anxiety and stress for both those with SEND and their parents or carers, around testing in the home was reported to negatively impact feelings of comfort and safety. Parents reported the ways in which these near-traumatic experiences could prompt distrust and damage familial relationships:

“The problem is once you’ve done it once, it breaks that trust you have with them. Like you’ve said, you mention COVID she’s off. She’s like ‘no because you’re going to pin me down’” (Parents Focus Group 7).

This was felt particularly acutely by the family of a young person who was non-verbal, the impact of a negative experience taking place at home caused significant barriers to engaging in testing:

“My kid can’t communicate so why would I do something to impact his mental health in a negative way when I don’t need to. My kid relies on me to keep him safe, and our home is a place he is safe” (Parent Focus Group 3).

Parents attempted to reassure children or young people by demonstrating a testing kit, in one case by swabbing soft fruits, to illustrate to their child that testing does not harm them:

“We tried to get my son to do a lateral flow. We were like practicing on pieces of fruit, cutting up strawberries, cutting up an orange, things like that, and we were like, look, this is all it does. It just wipes it. Well, then an orange came back positive, and the strawberry came back positive. Literally, he had a complete meltdown that this orange and this strawberry had come back positive. ‘I’m not eating them. They’ve got COVID’. He’s only just started eating fruit again” (Parents Focus Group 6).

The coincidental positive test result after using fruit as a demonstration unfortunately had a backfire effect and had a compounding lasting impact that damaged trust in another way, in this case promoting fear around eating fruit.

### Illusion of choice

Participants reported perceiving testing as so distressing, uncomfortable and even painful, meaning that a decision or choice to test was, in reality, invalid. Parents described an inevitability in declining to test as, in practice, testing was unfeasible due to the negative emotional and physical impact in the context of the needs of young people with SEND, therefore their ‘choice’ to test or isolate was never a genuine choice in reality:

“I tried once or twice, and he was hyperventilating until the point of being sick. I was just like, right, I’ve actually had to keep him off school, and say: ‘I can’t do the test’, and then do the ten days isolation and I literally had to keep him off, knowing that he didn’t have it, and they won’t let him in” (Parent Focus Group 6).

This was reported to be the case even when parents sought support from healthcare professionals throughout the pandemic:

“I asked for help and phoned 119, the hospitals, and literally everyone was like: ‘isolate them for 10 days’. Either isolate them or restrain them” (Parents Focus Group 7).

Participants reported that they felt isolation was the only feasible option as testing could only be realised via restraint, which was incredibly distressing for all involved, and this undermined families’ empowerment to make decisions.

### (Mis)Understanding of needs

Our participants often articulated occasions or examples where support was required in order to fully engage in testing, however they found that there was no or inadequate provision in these cases. Further to participants reporting that they had no choice but to isolate given the barriers they encountered with testing these experiences prompted perceptions that people with SEND had been overlooked in both testing guidance and practice, ultimately leading to a sense of disenfranchisement in addition to the negative emotional and physical experiences described above.

Parents reported struggling to access testing services for their child due to their needs not being met at testing centres. Some PCR testing services were situated in environments that provided a sensory overload for those with SEND, and parents reported having difficulties in managing the child’s emotions in these environments:

“When you go for like for a planned test at hospital and we had to go underground car park. It makes a noise when you go in, and she didn’t like that, and then you had to park up in this spot, and it felt claustrophobic, so she didn’t like that. And then this woman came that she didn’t know. She really didn’t like that, and then didn’t explain what she was going to do. She said: ‘right, come on, stop being silly’, and we’re like: ‘that’s not how you deal with her’” (Parents Focus Group 6).

Both the physical environment and the support provided by staff failed to make accommodations to support individuals with SEND in experiences reported by parents. Many parents in this project reported calling testing centres ahead of their visit to request support in accessing testing, unfortunately to find that no alternative measures were in place at the centres:

“I phoned our GP. Again, they were like: ‘no we can’t do anything. We’ll book you an assisted test’. Went to testing: ‘no we don’t do assisted tests for under 12s. No we can’t help you’. Went out of the car. Husband is scrambling around trying to pin a five-year-old in a car seat to get a test up his nose” (Parents Focus Group 7).

The lack of support at testing sites was reportedly exacerbated when staff there failed to understand that for families supporting their children with SEND, it may be the case that more than one adult will need to accompany them to a testing centre in order to fulfil the test:

“I don’t drive. You can’t have the siblings in the car. You can only take the child who you are testing, and they have to sit on the window seat. They were even being funny about that, because I wasn’t the driver. But he won’t have it done without me being there (Parents Focus Group 6).

It was also reported that the spectrum of special educational needs had been overlooked in its diversity and the potential considerations, which may need to have been made in order to support all to engage in testing. Particularly expressed was a sense that, for parents, the gravity of taking a test for those with SEND had been underestimated in practice:

“I think as well, the professionals don’t quite get the autistic aspects of it, and ‘it’s just a test’, do you know what I mean? Or even the school said to me: ‘could you just do a test on him?’ It’s like, you can’t come back without a test, and it’s like, hard to explain that. There was no autism awareness around that, if you know what I mean” (Parent Focus Group 6).

It was suggested that support could involve professionals in healthcare settings and in the community trained specifically to perform tests for people with SEND:

“For me, it would have been easier if the option was always there. If we could, for example, if we talk about solutions, if at every Boots [Pharmacy], there would be somebody who could administer this for special needs children, or special needs people” (Parent Focus Group 2).

The impact of having to plug the gaps parents felt to be present in the system during an already difficult time resulted in reinforced feelings of exclusion and a lack of representation for our parent participants and their families with SEND:

“There are no provisions made by the NHS or by the government for children like ours” (Parent Focus Group 7).

### Unmet communication needs

Participants highlighted that there was a lack of information aimed at those with SEND about COVID-19 testing, specifically an absence of tailored information about how to take the test that was suitable for those with SEND: “*it’s not autism friendly […] it’s complicated as well” (Parent Focus Group 6)*. Strategies and resources were said to be needed to help parents explain COVID-19 in the first instance and thus testing to their children with SEND. Parents suggested that having information that parents can tailor to the needs of their child would be helpful:

“The spectrum’s so vast […] it’s not going to be one size fits all with autism, is it?” (Parent Focus Group 6).

Parent participants reported that, in their experience, any information they received about COVID-19 testing was from other parents or guardians on social media, and not from health professionals or the government:

“I’ve had more info provided to me by other parents who’ve found snippets of things so I’m on quite a few Whatsapp groups with parents and they’ll just send stuff” (Parent Focus Group 1).

Parent participants also reported that for their children with physical disabilities, a lack of interpreters on TV reports meant social media was sometimes their only source of information for their child to access: *“He couldn’t understand what COVID-19 was and he was getting his information online because the news didn’t always have an interpreter” (Parent Focus Group 5).* In discussions with participants with SEND, there was acknowledgement that: “*sometimes there’s fake news all over Facebook and stuff” (SEND Focus Group 2)* and an appetite expressed to receive information from trusted sources, for example medical professionals delivered in-person: “*I would prefer a doctor or like a specialist or something […]I want someone coming to talk to you about it” (SEND Focus Group 2).* The call for more information from trusted sources was echoed by parents:

“That information I would really expect to get it from the medical facilities so that they would be able to tell me which exact test will be able to help me and help my child at the same time” (Parent Focus Group 4).

For those with physical disabilities, parents and guardians suggested that testing centres were not equipped to deal with individuals who needed particular support. Instances when testing centres did not always have an interpreter available were reported and due to mask-wearing, those with hearing impairments were unable to access these centres:

“He’s not always able to access places or people don’t have the support in place to deal with deaf people. People wear masks and there’s no interpreters available so what would he do? He would struggle” (Parents Focus Group 5).

For those with visual impairments, parents reported that testing instruction were not made available in a format that was suitable for their children’s needs. Parents suggested that audio information or instruction booklets that were in braille were needed so that their child was not excluded, and they were able to share information and indeed test results, to complete the test appropriately:

“My worry is that for her, she won’t be able to see them because she’s blind. Unless she has someone there to take them with her. So, that’s going to be a challenge for her. Taking the test will be easier for her if there’s audio instructions or rather braille instructions in the testing kit so that she can be able to access them on her own” (Parent Focus Group 4).

### Experts in their own experience

The parent participants in this project demonstrated a wealth of knowledge and experience of how to adapt in situations to facilitate and meet the needs of their child. Whilst the COVID-19 pandemic represented unchartered territory for all, parents shared methods which they employed to, as best they could, adhere to the testing guidelines at the time, whilst maintaining much-needed consistency for their child with SEND.

#### Importance of routine

Routine was discussed by parents as crucial for many young people with SEND, especially in cases where young people were neurodivergent. From experiences reported during this project, the sentiment families expressed pointed to feelings that the COVID-19 testing requirements, from a systemic viewpoint, overlooked the impact a disrupted routine can have on people with SEND.

Changes to those routines are planned for, sometimes weeks in advance by families and in discussions, parents reflected on the impact changes throughout the pandemic generally had on routine:

“When they put lockdown […] throughout the country […] they didn’t think about our children. They locked us all down. You can’t attend school. You can’t do this, that, or whatever. But that changes our kids’ routines […] we’re all stuck at home trying to figure out how to calm these kids down, put a new routine in place […] Then they’re like: ‘right, the country is opening back up as of this day’ […] If you’re lucky you get a week’s notice. If not, you’ve got a few days’ notice. You can’t change that child’s routine within a few days. It’s just don’t work” (Parents Focus Group 7).

The importance of an undisrupted routine for those with autism especially was discussed by parent participants. Specific to the impact of testing on routines, parents reflected on the negative impacts short notice changes to routine can have when supporting their child to engage in testing:

“If you are autistic […] a change of plans like that is even harder than for us, then this whole thing becomes very dramatic. We could not make it much easier” (Parent Focus Group 2).

“Especially the child with special needs. Because his reaction was so much more, in some ways. He became quite fearful of it. And once he gets a pattern of behaviour like that, once he gets this anxiety about just anticipating it, that’s a hard pattern to break with him” (Parent Focus Group 2).

Parents, once again, shared a wealth of knowledge and expertise in using techniques to support their children with SEND to engage in testing and manage their expectations when testing represented a disruption to the status quo:

“Whenever we travelled or whenever we planned something, like a birthday party, visiting people, we started saying that ‘we are trying to do it, we are trying to have a party. If the test is negative, we can have it’. ‘We are trying to go on holiday, if the test is negative’, instead of saying ‘we are going’” (Parent Focus Group 2).

Part of the importance of routine is needing time to adjust to a new routine. Parents shared examples of necessary preparations they made in the lead up to testing their children. This was to attend to their children’s needs for consistency, particularly for neurodivergent children, and minimise further anxiety, which they may already have been experiencing on account of significant changes to daily life during the pandemic. Parents reported experiences being easier because they “built up to it” (Parent Focus Group 1) or using techniques such as “role play and work through it” (Parent Focus Group 6) and using music to create a calming atmosphere directly before testing. As discussed earlier, there was an example shared by a parent attempting to demonstrate to their child the harmlessness of the test by performing it on fruits. Whilst this had a backfire effect, preparation and demonstration was reported to have positive impacts on engaging in testing for those with SEND. Whilst most of the experiences reported to us were preparations performed by parents themselves, an example was shared where staff at a children’s hospital had helped to prepare a young person with SEND for their test:

“I seem to be the exception but all three COVID tests at the children’s hospital, they were amazing with him. They showed it to him. They explained what they were doing” (Parent Focus Group 6).

This positive experience of testing at a children’s hospital was attributed to preparation and demonstration. These examples point to the wealth of experience and expertise these families have in supporting their children with SEND, in particularly difficult circumstances and point to knowledge of support methods which could be harnessed in approaches, both guidance and practice, to public health testing in future.

#### Parents filling capability gaps

Whilst recognising and expressing a keenness to follow government guidance or recommendations, parents reported having to resort to flexible interpretations or practices, which enabled their families to continue to function. This included working from home or, when this was impossible, taking annual leave when testing to access school or other events failed. Parents also reported buying tools to support the administering of tests, such as sensory blankets.

Parents reported the need to interpret and translate guidance, depending on the needs of their children. Many reported that the guidance, when conducted in practice by a health professional rather than when parents administered at home, was done with more pragmatic flexibility than “the instructions made you feel you had to” (Parent Focus Group 2), that is: inserted in nose until resistance was felt, swabbing tonsils and avoiding tongue. Resulting in feeling that instructions or guidance “[…] did make it seem quite a bit more difficult than it appeared when a professional did it” (Parent Focus Group 2).

Others described informally triaging their child’s symptoms to decide if a COVID-19 test was needed, as testing temperature, for example, was less invasive and problematic:

“I probably test him for his temperature more than actually shoving a test up his nose now and just hope for the best. But visiting family’s going to be the difficult thing and visiting older relatives. That’s where you get worried. We will be testing at those points” (Parent Focus Group 1).

There was acknowledgement, however, about risk perception and particularly for impact on more vulnerable family members. Parents acknowledged that in practice, making judgement calls was based on a balance of risk based on the symptoms displayed, in this case a temperature, against the vulnerability of family members. Making these judgement calls was deemed a necessity by these parents in the context of the impact of testing and inflexibility of guidance in practice.

This judgement as to when a COVID-19 test was necessary or indeed feasible was expressed by others with comments such as “we did the best we could” (Parent Focus Group 1) and a recognition of the need to be honest with these interpretations of guidance:

“In some ways we tested as best we could, but we didn’t test him every day and I was very honest with school” (Parent Focus Group 1).

Parents of children and young people with SEND have a clear understanding of their needs and how to cater to them, as shown in the many examples described above and reinforced below, with the call to understand these experiences of testing from the perspective of those with additional needs:

“Thinking of it from the perspective of a special needs kid, I think that it has to be done in a unique way that understands the needs of the kid, first of all, that understands the need of the kid and in a way that doesn’t necessarily stigmatise the kid. Because, first of all, this is a kid that doesn’t really understand what’s really going on. As a parent I have to take care of the best interests of my kid” (Parent Focus Group 3).

## Discussion

Our study has demonstrated people with SEND and their families and carers had difficult and negative experiences of COVID-19 testing during the COVID-19 pandemic. These experiences have had lasting effects and point to the need for greater support for and understanding of the variety of needs individuals may have, in order to support them to engage in public health interventions. For people with SEND and those who care for them, opportunities to engage in COVID-19 testing were hampered and limited by both physical and emotional barriers, which were underpinned by a misunderstanding of how testing may need to be administered in practice with those with special educational needs or disabilities, compared to the wider population. This misunderstanding resulted in parents and carers finding other ways to meet the needs, as they relate to testing, of their children with SEND by remaining adaptive and flexible.

The effort parents expressed when trying to account for an unresponsive system reflects previous research that demonstrates how managing chronic illness and disability is often experienced as a type of ‘work’. Building on Strauss’s concept of ‘articulation work’, which is encountered when managing chronic illness [38], Shearn and Todd [39] argue that this type of work is also synonymous with the type of ‘work’ parents of people with SEND experience as part of their daily lives. Articulation work refers to the overarching labour, such as coordinating, prioritising, and arranging, which are needed to enable everyday tasks and activities to be engaged with. For parents of people with SEND, this articulation work also involves trying to manage incompatible time frames, where the need for temporal flexibility to complete tasks clashes with restrictive and inflexible externally imposed timescales [39]. This challenge of incompatible timeframes was experienced by parents in our study, articulated through their discussions around the challenges of following public health guidelines regarding testing. This involved a change in their child’s routine and how the introduction of regular testing was experienced as an abrupt and significant change to daily life, which was not compatible with their normal routine.

Examples heard from parents about the need to provide demonstrations of how to perform the tests were prompted by the negative, sometimes painful and anxiety-inducing aspects of LFD and PCR tests for our participants with SEND. Families have described using fruit to demonstrate that testing can be pain-free, however as outlined above, there were unintended consequences associated with this. More broadly, the need to resort to this type of demonstration points to the requirement for more resource in explaining the use of tests to those who may need more assistance in understanding the process of testing and acts as a lesson for how to better engage those who may need more support to be included more sensitively in testing programmes in the future [4, 5, 8, 10, 13]. Reflections on communication in SEND-specific or accessible ways raised in focus group discussions begins to highlight the importance of considering the needs of users fully when inviting them to engage in public health interventions [5, 10]. This is echoed by other research calling for greater “collaborative practice, putting families in the driver’s seat, and ensuring support services are designed to foster engagement and be responsive to families’ needs” [5, 10, 13, 40].

The parents involved in this study showed a deep understanding of how and why the testing offer needs to be adapted and therefore more accessible to those with SEND. Experiences which did not meet the needs of their children or young people with SEND reinforced feelings of exclusion for all participants [4, 5, 10, 13]. Of course, changes to daily life at short notice can be expected to an extent during a pandemic, however specifically around testing requirements an acknowledgement of the impact such changes can have on people with additional needs was felt to be, from our participants’ experience, overlooked in the public health guidance and practice [5, 13]. In response to the perceived gaps in guidance or provision which left their needs unmet, parents and families discussed ways in which they had to be adaptive, flexible, and even rationalise an interpretation of guidance provided. Parents further reported experiences of adjusting their daily lives to facilitate their children’s engagement with testing. Methods used to address these system deficits included changes to language used to reflect the necessary flexibility in routine in a measured way [8, 11, 41]. These findings of experiences of pressure on parents to “get it right” [40] are borne out in wider literature, which highlights the impact such stressful experiences can have on the parent-child relationship, as well as feelings of parental guilt [10, 40].

The unmet needs in cases where families of those with SEND tried to engage appropriately in testing resulted in experiences of frustration and ultimately disenfranchisement and exclusion [5, 13]. Parents have advocated for an approach to testing, in both guidance and practice, which is underpinned by a holistic understanding of the spectrum of SEND [5, 13]. Barriers to testing reported above were attempted to be overcome by the participants, but ultimately proved insurmountable in most examples shared. Both in guidance and in practice, the testing system was perceived to be unreflective of young people with SEND and their parents as experts in their own experience, who have a deep understanding of how and why the testing offer needs to be adapted and therefore more accessible [5, 13, 40, 42].

Our study highlights how parents have a deep understanding of their child’s needs; they have had to become experts. Becoming an expert and advocating for their children is familiar to parents of children with SEND, particularly in relation to the complexities of medical care and appointments [43]. It is important to note that becoming an expert in this way is a considerable amount of work and families have called for this effort to be better recognised by government during the pandemic [5]. While developing expertise can be empowering, previous research has also demonstrated that one of the unintended consequences of the sharing of this knowledge developed is the additional labour it entails [44].

This notion of engagement with COVID-19 testing being ‘work’ reflects previous research on disability and illness, where people with chronic conditions described the work they had to perform as illness related (managing symptoms), biographical (coming to terms with a new sense of self) and everyday life (adjusting to the practicalities of everyday life) [45]. Further to this, research with women on their chronic condition, specifically endometriosis, demonstrated how women experience patient expertise as a form of work. Referred to as the “third shift”, in reference to the paid and unpaid work women are often already engaged in, this study highlights the overwhelming amount of often complex and contradictory information that needs to be synthesised. This is in addition to a sense of always being “on duty”, which resulted in women feeling a personal and moral failure if they were unable to effectively manage their condition [46]. In this context, our study also serves as a reminder of the importance of respecting the expertise that comes from lived experience, but balancing that expertise with a sense of care, understanding and acknowledgement provided by authorities, so people with SEND and those who care for them feel empowered, not overwhelmed [5, 10].

These findings contribute to the growing body of literature which captures the experience of those with SEND and their families during the COVID-19 pandemic [5–8, 10, 40, 47]. It also adds to the wider literature on embracing lived experience in research on health, illness and chronic conditions particularly for those with additional needs or disabilities [5, 10].

### Limitations

This research has some limitations which should be acknowledged. As referenced above, the use of focus groups with those with SEND has some limitations and alternatives such as more participatory methods have been suggested as more effective and engaging for individuals with a range of needs [28]. However, in the case of this project the data collection methods remain appropriate due to the sometimes-sensitive nature of the experiences shared by participants and the need to capture the nuance and depth of experience of our participants [25, 29–31]. Additional limitations relate to the sample size (*n* = 29) and whilst the research was situated to the North of England, testing practices and procedures were applicable across England and in similarly mirrored across parts of Europe. In addition, the participants were predominantly neurodivergent individuals. While this reflects the experiences of an important part of the SEND population, the findings may not fully capture the experiences and needs of individuals with other types of special educational needs or disabilities, including those with sensory, physical or complex learning disabilities. Finally, as detailed in the methods section above, it should be acknowledged that data collection was conducted by one member of the research team who left the team during the project, therefore the data were analysed by the two remaining members of the project team. Given this, the two remaining team members took the time to ensure thorough understanding of the data and its context, with handover meetings and time spent iteratively developing a coding framework from the data, resulting in refinement of codes and ultimately themes.

#### Recommendations

In order to harness the findings of this project and others [5, 8, 10, 11, 13, 40, 41], the following is recommended for the development of guidance and practice:

1. Incorporate the lived experience of people with SEND and those who care for them using behavioural and social science approaches when developing testing approaches in future. This could go some way to addressing the gaps parents and families in this research felt the need to plug themselves [13, 5, 10, 41].
2. Acknowledge, learn from and use this expertise of experience to develop guidance using co-production approaches, which are inclusive and embody the experience of those with SEND and their families [5, 8, 10, 40, 42]. These approaches should recognise the contributions of the lived experience experts, particularly for the additional labour sharing these experiences can incur [5, 44].
3. Guidance to testing and broader health protection approaches should reflect the diversity of experience of SEND, the spectrum of needs and recognise this community is non-homogenous, therefore guidance should seek to appreciate the need for flexibility [5, 10, 13].
4. Based on more inclusive guidance, testing in practice should be resourced in order to be truly accessible for the spectrum of needs for those with SEND and their families. This includes supporting face-to-face practitioners to understand and attend to the diversity of needs of people with SEND in testing [5, 10, 13].

## Conclusion

“I genuinely think during the whole pandemic, our children are the forgotten children […] I really do. I think during this whole thing they were literally the forgotten children. Regarding everything regarding COVID, not just testing” (Parent Focus Group 6).

The lack of inclusive COVID-19 testing and support left people with SEND and those who care for them feeling ignored and frustrated. In future, policy and guidelines that include and reflect the needs of those with SEND are needed to better improve the practice of health protection testing initiatives [5, 8, 10, 13, 40, 47]. In addition, where work has been done to evaluate alternative methods of testing, this needs to be more effectively communicated. The results of this project point to an absence of SEND-specific or accessible ways in which engagement to testing has been supported in practice and highlights the importance of considering the needs of users fully when inviting them to engage in public health interventions. Alternative methods of testing should be made available for those with SEND to enable equitable access to services, helping to reduce health inequalities. The adverse elements of COVID-19 testing experience were felt more acutely, we argue, because of a cyclical misunderstanding of needs of people with SEND present in the expectations from health systems in the cases of our participants. This acted as an undercurrent to much of what was reported in this project, resulting in feelings of disempowerment and marginalisation [8, 13]. Aspects of these experiences have an impact not only at the time of data collection, but also in terms of legacy of public health, particularly health protection programmes. The experiences shared in this research point to the wealth of expertise these families have in supporting their children with SEND and to a vast knowledge of support methods, which could be harnessed in approaches, both guidance and practice, to public health testing in future. These findings sit in the context of a socio-historic landscape of SEND, typified by definitional disputes and homogenisation as vehicles for needs being overlooked and unmet [5–7, 13, 27], alongside the compounding effect of the pandemic more broadly [5, 8, 10, 11, 13, 40, 41].

This paper has presented an in-depth exploration of the testing experience of young people with SEND and those who care for them and its impact. The participants in this project are highlighted as experts in their own experience. Deferring to their knowledge and experience, capturing and learning from such will have positive consequences for the development of public health and health protection guidance in future.

## Supplementary Information

Supplementary Material 1.

Supplementary Material 2.

## Data Availability

Data is available on request from the lead author. Materials have been published with the study protocol on Open Science Framework, available here: https://osf.io/f47dx.

## Abbreviations

ADHD: Attention Deficit Hyperactivity Disorder
ASD: Autism Spectrum Disorder
LD: Learning Disability
LFD: Lateral Flow Device
ND: Neuro-Diverse
PCR: Polymerase Chain Reaction
REGG: Research Ethics and Governance Group
SEND: Special Education Needs and Disabilities
UK: United Kingdom
UKHSA: UK Health Security Agency
