# Supplementary material for: “Our children are the forgotten children”: lived experiences of COVID-19 asymptomatic testing in young people with special educational needs and disabilities and those who care for them
Source: BMC Public Health. 2026 Jul 28;26:3037. doi: 10.1186/s12889-026-28743-w (PMC13688956; doi:10.1186/s12889-026-28743-w)
Supplement: Supplementary file 2 [file 12889_2026_28743_MOESM2_ESM.docx]

**Supplementary file 2: Easy Read Information Sheet**

**Information Sheet**

**Experiences of regular COVID-19 testing in people with Special Educational Needs and Disabilities.**

[Name of researcher] from UK Health Security Agency would like your help.

**Picture of researcher conducting data collection**

[Name of researcher] would like to have a short discussion (around 15-20minutes) about how you feel about COVID testing.

To do this, [name of researcher] would like to hear about your experiences of testing.

This can be done either by [name of researcher] asking you a few questions or reading a story together.

Don’t worry you won’t be on your own! An adult that you know from [insert organisation] will be with you throughout.

The discussion will take place at [insert organisation] at a time that suits you.

You can stop the discussion at any time if you don’t want to continue.

[Name of researcher] will record the discussion on a Dictaphone. This is so she can spend time talking with you and it helps [name of researcher] remember what has been said.

Whatever you say is completely anonymous, that means that no one will know that is has been said by you.

[Name of researcher] will share the findings from the research with you. The findings could help make testing easier for other people.

Are you happy to take part?
