# Supplementary material for: “Our children are the forgotten children”: lived experiences of COVID-19 asymptomatic testing in young people with special educational needs and disabilities and those who care for them
Source: BMC Public Health. 2026 Jul 28;26:3037. doi: 10.1186/s12889-026-28743-w (PMC13688956; doi:10.1186/s12889-026-28743-w)
Supplement: Supplementary file 3 [file 12889_2026_28743_MOESM1_ESM.docx]

**Supplementary file 1: Flashcards**

**Storyboard of Testing Journey:**

**Mood cards / emotion faces:**
